# Diagnostic and Prognostic Markers for Pancreatitis and Pancreatic Ductal Adenocarcinoma

**DOI:** 10.3390/ijms25126619

**Published:** 2024-06-16

**Authors:** Havish S. Kantheti, Michael A. Hale, Shreoshi Pal Choudhuri, Huocong Huang, Xu-dong Wang, Yalda Zolghadri, Giulio Innamorati, Sai Prasada Rao Manikonda, Naviya Reddy, Sarthak Reddy, Rahul K. Kollipara, Valbona Lumani, Luc Girard, Yakov Bezrukov, Pavel Demenkov, Raymond J. MacDonald, Rolf A. Brekken, Yonghao Yu, Thomas M. Wilkie

**Affiliations:** 1 Department of Pharmacology, UT Southwestern Medical Center, 6001 Forest Park Drive, Dallas, TX 75390, USA; havish.kantheti@tamu.edu (H.S.K.); michael.hale@utsouthwestern.edu (M.A.H.); shreoshi.palchoudhuri@utsouthwestern.edu (S.P.C.);; 2 Cancer Discovery (CanDisc) Group, UT Southwestern Medical Center, 6001 Forest Park Drive, Dallas, TX 75390, USA; yalda.zolghadri@univr.it (Y.Z.);; 3 Texas A&M School of Engineering Medicine, 1020 Holcombe Blvd, Houston, TX 77030, USA; 4 Department of Neuroscience, UT Southwestern Medical Center, Dallas, TX 75390, USA; rahul.kollipara@utsouthwestern.edu; 5 Hamon Center for Therapeutic Oncology Research, UT Southwestern Medical Center, Dallas, TX 75390, USA; huocong.huang@utsouthwestern.edu (H.H.); luc.girard@utsouthwestern.edu (L.G.); rolf.brekken@utsouthwestern.edu (R.A.B.); 6 Department of Biochemistry, UT Southwestern Medical Center, 5323 Harry Hines Blvd, Dallas, TX 75390, USAyy3213@cumc.columbia.edu (Y.Y.); 7 Department of Surgical Sciences, Dentistry, Gynecology and Pediatrics, University of Verona, 37126 Verona, Italy; giulio.innamorati@univr.it; 8 Department of Biosciences, Rice University, Houston, TX 77005, USA; 9 Cogia AG, Poststr. 2-4, 60329 Frankfurt, Germany; y.bezrukov@cogia.de (Y.B.);; 10 Department of Molecular Biology, UT Southwestern Medical Center, Dallas, TX 75390, USA; 11 Department of Surgery, UT Southwestern Medical Center, Dallas, TX 75390, USA

**Keywords:** PDA early detection, scRNAseq screen, mouse KIC, human PDA cell lines, MS mass spec, early epithelial markers, temporal markers of PDA progression, cancer and stromal cell expression, diagnostic and prognostic markers

## Abstract

Diagnostic markers are desperately needed for the early detection of pancreatic ductal adenocarcinoma (PDA). We describe sets of markers expressed in temporal order in mouse models during pancreatitis, PDA initiation and progression. Cell type specificity and the differential expression of PDA markers were identified by screening single cell (sc) RNAseq from tumor samples of a mouse model for PDA (KIC) at early and late stages of PDA progression compared to that of a normal pancreas. Candidate genes were identified from three sources: (1) an unsupervised screening of the genes preferentially expressed in mouse PDA tumors; (2) signaling pathways that drive PDA, including the Ras pathway, calcium signaling, and known cancer genes, or genes encoding proteins that were identified by differential mass spectrometry (MS) of mouse tumors and conditioned media from human cancer cell lines; and (3) genes whose expression is associated with poor or better prognoses (PAAD, oncolnc.org). The developmental progression of PDA was detected in the temporal order of gene expression in the cancer cells of the KIC mice. The earliest diagnostic markers were expressed in epithelial cancer cells in early-stage, but not late-stage, PDA tumors. Other early markers were expressed in the epithelium of both early- and late-state PDA tumors. Markers that were expressed somewhat later were first elevated in the epithelial cancer cells of the late-stage tumors, then in both epithelial and mesenchymal cells, or only in mesenchymal cells. Stromal markers were differentially expressed in early- and/or late-stage PDA neoplasia in fibroblast and hematopoietic cells (lymphocytes and/or macrophages) or broadly expressed in cancer and many stromal cell types. Pancreatitis is a risk factor for PDA in humans. Mouse models of pancreatitis, including caerulein treatment and the acinar-specific homozygous deletion of differentiation transcription factors (dTFs), were screened for the early expression of all PDA markers identified in the KIC neoplasia. Prognostic markers associated with a more rapid decline were identified and showed differential and cell-type-specific expression in PDA, predominately in late-stage epithelial and/or mesenchymal cancer cells. Select markers were validated by immunohistochemistry in mouse and human samples of a normal pancreas and those with early- and late-stage PDA. In total, we present 2165 individual diagnostic and prognostic markers for disease progression to be tested in humans from pancreatitis to late-stage PDA.

## 1. Introduction

Reliable markers and effective therapeutics are needed for people at risk of developing or already afflicted with pancreatic ductal adenocarcinoma (PDA) [1]. For PDA, surgical intervention is currently the only effective treatment for the extension of lifespan, and only 20% of individuals are diagnosed with pancreatic cancer early enough to be surgical candidates [2,3]. New therapeutics are needed for late-stage patients, but they are the most refractory to treatment. Our approach is to focus initially on personalized medicine for the two classes of patients most likely to benefit at this time: (1) those with PDA who survive resection and are likely to succumb to recurrent cancer without further treatment; and (2) those at a high risk of developing PDA. Risk factors for PDA are pancreatitis (chronic or hereditary), smoking, obesity, and adult-onset type 2 diabetes (T2D), as well as having inherited cancer risk genes [4]. Some of the most effective therapeutics for early-stage disease may also help late-stage patients. 

Prior screening of late-stage tumors has been used to analyze bulk mRNA expression of mouse and/or human samples to describe distinct types of PDA and has identified multiple diagnostic markers [5,6]. A comparison of gene expression profiles show that PDA tumors in KIC mice are closely related to about 20% of PDA samples in the TCGA database PAAD (oncolnc.org) and provide a good potential source of markers for at least a subset of patients. KIC mice express oncogenic Kras^G12D^ and inactivate the tumor suppressor Cdnk2a in all pancreatic cell types, including islet, duct, acinar, and cancer cells in PDA. KC mice, which express wildtype Cdnk2a, develop PDA slowly, whereas KIC mice are a particularly aggressive mouse model of PDA [7] and a rich source of potential markers. An advantage of interrogating KIC mice is that early markers can be collected from young mice and late markers from older mice. 

To identify diagnostic and prognostic protein markers for all stages of pancreatic disease, we first isolated protein from PDA tumors in the mouse models of PDA, KC and KIC [7] and used mass spectrometry (MS) to identify differentially expressed proteins [8,9]. To enrich our analysis of likely fluid-born protein markers, we specifically used MS to screen for proteins preferentially secreted into conditioned media by PDA cancer cells compared to normal duct cells. We then queried scRNAseq data from KIC mice [10] to identify differentially expressed markers in cancer and associated stromal cell types, particularly focused on candidate approaches that would reveal early-stage markers in transition from benign neoplasia to cancerous lesions. 

Because oncogenic Kras is an early driver of 90% of human PDA cases [3], we screened the Ras pathway and cancer-associated genes but found few differentially expressed early markers. Therefore, we expanded our search for early markers in an unsupervised screen of early- versus late-KIC neoplasia and several candidate pathways known to impact initiation and progression in PDA. 

As pancreatitis is a risk factor for progression to PDA in the context of oncogenic Kras mutation [11], we screened for pancreatic genes that were differentially expressed in mouse models of pancreatitis, including caerulein treatment of normal mice [12] and the pancreas of knockout mice with the acinar-cell-specific deletion of one of four differentiation transcription factors (dTFs) (*Ptf1a*, *Nr5a2*, *Foxa2*, or *Gata4*) and the double knockout of *Foxa2* + *Gata4* [13,14,15]. Caerulein is a mimetic of the Gq-coupled agonist CCK [16] which immediately provokes calcium signaling, followed by the down regulation of the dTFs, dedifferentiation of acinar cells, and AP-1- and NFkB-mediated cytokine signaling during tissue repair [11,12,17,18]. Therefore, we also screened calcium and innate immune pathway genes in scRNAseq samples from early- and late-stage KIC neoplasia and tumors. 

Prognostic genes associated with worse or better prognoses in PAAD (the abbreviation for PDA used on oncolnc.org) were identified among the differentially expressed diagnostic markers we obtained from our initial screens. Therefore, we tested all the genes that were significantly associated with either worse or better prognoses in PAAD for differential expression in cancer and stromal cells. In aggregate, we identified 2165 individual diagnostic and prognostic markers (Supplementary Table S1) and devised an approach to validate them in mouse models of PDA (KIC) and human tumor samples. 

## 2. Results

### 2.1. Single-Cell RNAseq Analysis of PDA in KIC Mice

**Figure 1:**
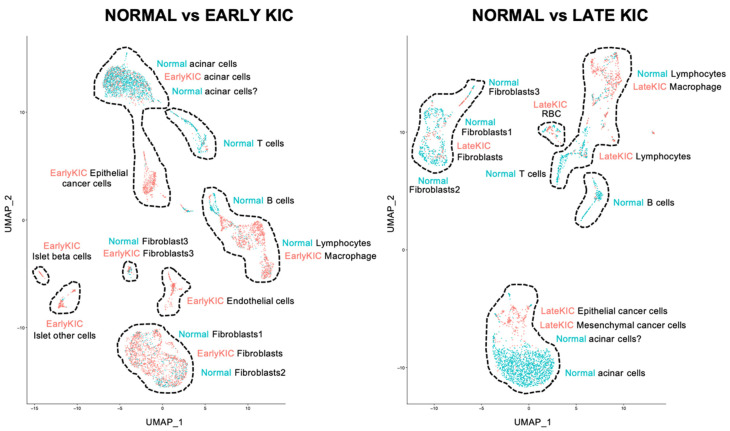
PDA cell types identified in early- and late-stage KIC compared to wildtype pancreata. t-distributed stochastic neighbor embedding (tSNE) plot of combined cells in normal pancreas (2354, comprising 8 distinct cell populations pooled from 2 mice), early-stage KIC lesions (3524, 8 cell types pooled from 2 mice), and late-stage KIC tumors (804, 6 cell types pooled from 3 mice). Normal “acinar cells ?” (referred to as “acinar 2” in Figure 2 and Figure 3) express cell stress markers.

PDA is among the most lethal cancers because it is detected late, there is no cure, and effective treatments extending patients’ lifespan are lacking. Our initial effort to find new markers for its early detection and diagnosis was to screen for differentially expressed genes in scRNAseq samples from normal pancreata vs. early- or late-stage KIC tumors. scRNAseq datasets (Figure 1 [10]) were used to compare the cell-type identity and relative abundance of the mRNA encoding candidate marker proteins and genes expressed in early-stage PDA (pancreata from 40-day-old KIC mice) or late-stage PDA (pancreatic tumors from 60-day-old KIC mice) vs. a normal mouse pancreas (40 days old). The predominant cell types in the pancreata from the normal and early- and late-stage KIC mice (Figure 1) were identified by cell-type-specific markers described in Hosein et al. [10] (Supplementary Figure S1A) and corroborated by additional pancreatic markers (Supplementary Figure S1B, Supplementary Table S1_Y_Cell ID; 41 genes total). 

### 2.2. Cell-Type Expression of Diagnostic PDA Markers 

**Figure 2:**
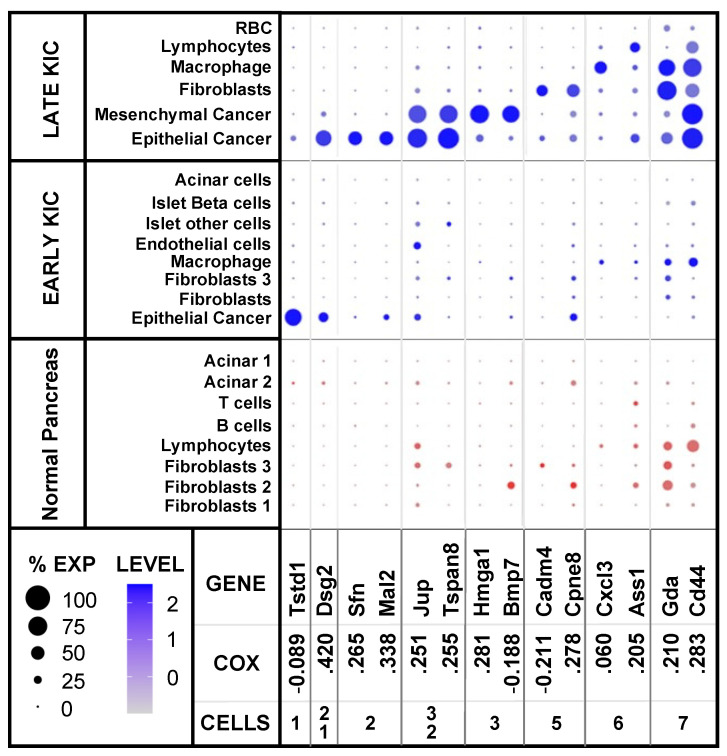
Quantitative dot plots (QDPs) of cell-type distribution and levels of exemplary PDA markers in mice. PDA markers identified by MS in mouse KIC tumors or conditioned media from human PDA cell lines were further characterized by QDP analysis of scRNAseq from PDA tumors in KIC mice at 60 days (Late KIC) or 40 days (Early KIC) or from a normal pancreas at 60 days [8]. Representative differentially expressed genes in KIC tumor cell types compared to normal pancreas were detected in the following: (1) Epithelial cancer cells in early-stage KIC mice; (2) Epithelial cancer cells in late-stage KIC mice; (3) Mesenchymal cancer cells in late-stage KIC mice; (5) Fibroblast classes 1, 2 and/or 3 (F1, F2 or F3) in early- and/or late-stage KIC mice; and (6) Hematopoietic cells (macrophage, T cells, B cells or lymphocytes). (7) Markers were preferentially expressed in many cell types in KIC tumors compared to normal pancreas. Cox coefficients measure the association between mRNA expression level and patient survival outcome (oncolnc.org). The percentage (%) of cells expressed within a given cell type is represented by the size of the dot; an increased color intensity (log2) correlates with a higher level of expression (blue dots, tumor cell types; red dots, normal pancreas cell types).

The gene sets of the candidate markers, which are described below, were queried against scRNAseq samples (Figure 1) to identify the cell-type specificity of differentially expressed diagnostic and prognostic genes in PDA. We queried scRNAseq in a normal pancreas compared to early- and late-stage PDA tumors in KIC mice using quantitative dot plots (QDPs) to identify the cell-type expression of candidate genes (listed in Supplementary Table S1). Representative diagnostic markers are shown in Figure 2. The cell-type gene expression identified in our violin plots (Supplementary Figure S2A) and the QDP (Figure 2) were always in agreement (for comparison, see Supplementary Figure S2B, ITGA3).

Six cell types yielded a preponderance of the markers that were preferentially expressed in the cancer and tumor stroma. Differentially expressed genes were specifically expressed in three cancer cell types: epithelial cells in early-stage KIC (Figure 2, Tstd1), and in late-stage KIC epithelial (Figure 2, Sfn and Mal2) and mesenchymal cells (Figure 2, Hmga1 and Bmp7). Additional genes were expressed in the epithelial cells of both early- and late-stage KIC (Figure 2, Dsg2) or in both the epithelial and mesenchymal cells in late-stage KIC (Figure 2, Jup and Tspan8). This suggested the temporal control of cancer gene expression progressing from early epithelial to late mesenchymal cancer cell types. Other genes were preferentially expressed in the stromal cells of late stage-KIC tumors: fibroblasts (Figure 2, Cadm4 and Cpne8), macrophages (Figure 2, Cxcl3) and lymphocytes (Figure 2, Ass1). Some genes were broadly expressed in cancer and stromal cell types in late-stage KIC tumors (Figure 2, Gda and Cd44). 

The candidate markers we describe were identified using four approaches: (1) an unsupervised screening of genes preferentially expressed in the mouse PDA tumors (Supplementary Table S1_1C); (2) differential mass spectrometry (MS) to identify proteins of conditioned media from human cancer cell lines or mouse tumors (Supplementary Table S1_2D–2F); (3) signaling pathways that drive PDA, including the Ras pathway, calcium signaling and known cancer genes (Supplementary Table S1_3G–3T); and (4) genes whose expression is associated with poor or better prognoses (PAAD, oncolnc.org; Supplementary Table S1_4U and 4W). The cell-type distribution of 1230 genes preferentially expressed in cancer or tumor stomal cells is in Supplementary Table S1; 33 (3%) of these genes are restricted to early epithelial cancer cells, and 148 (12%) are first expressed here. The complete list of all 2165 differentially expressed genes in cancer and/or stromal cells in the PDA tumors of the early- and late-stage KIC mice is in Supplementary Table S1, column B (ST1_1B, Groups 1-4). These genes exemplify markers for PDA progression from early-stage epithelial to late-stage mesenchymal cancer and stroma.

### 2.3. Screenings of Pathways and Processes Implicated in PDA

The cell-type expression of candidate marker genes in the early- and late-stage PDA tumors in the KIC mice described below is summarized in Table 1 (gene names and cell-type expression are listed in Supplementary Table S1).

### 2.4. Candidate Gene Identification

#### 2.4.1. Approach (1): Unsupervised Screening for Differentially Expressed PDA Genes in KIC Mice 

The unsupervised screening was conducted for differentially expressed genes in scRNAseq samples from a normal pancreas vs. early-KIC or late-KIC tumors. A total of 503 differentially expressed genes were identified, primarily in epithelial or mesenchymal cancer cells in late-stage KIC tumors (Table 1; gene names are in Supplementary Table S1_1C). To help identify the best candidates for diagnostic markers in patient samples among these 503 genes and to find additional markers, we undertook a series of candidate gene searches among the proteins secreted by the PDA cells and pathways known to impact the initiation and progression of PDA. 

#### 2.4.2. Approach (2): Mass Spectrometry Identification of Human and Mouse PDA Proteins 

Secreted proteins are more likely than intracellular proteins to be useful markers and can be detected in the blood, stools, or pancreatic juice. To find new markers for early detection and diagnosis that are most likely to be secreted, we used mass spectrometry (MS) [8,9] to identify the proteins that were differentially expressed in conditioned media from five human PDA cell lines (ASPC-1, PANC-1, PL-45, MIA-PaCa-2 and BxPc-3; 3963 unique proteins) compared to a normal duct cell line (HPNE; 3860 proteins). Each PDA cell line expressed distinctive sets of secreted proteins as well as proteins in common with one or more of the other PDA cell lines (Supplementary Figure S3); 847 secreted proteins were expressed ≥4.5× more in human PDA cells than in HPNE cells (Supplementary Table S2), and 187 of these were differentially expressed specific cell types in KIC tumors compared to a normal pancreas (Table 1; gene names are in Supplementary Table S1_2D), but only 16% were expressed in early epithelial cells. There was a correlation of human PDA cell line mRNA and secreted protein abundance, with a few exceptions (Supplementary Figure S4), indicating RNAseq was an informative data set to identify differentially expressed protein markers. 

PDA from the solid tumors dissected from the KIC mice (60 days old) and samples for acinar-to-ductal metaplasia and early-stage PDA neoplasia were dissected from KC mice (at 40 days) and compared to normal pancreata from adult mice (60 days). MS identified approximately 3750 proteins in each sample (Supplementary Figure S5). The normal pancreata preferentially expressed digestive enzymes and regulatory pathway proteins that maintain acinar cell identity [19]. In contrast, candidate PDA markers were among the 200 mouse proteins expressed at least 4.5-fold higher in early- or late-stage PDA compared to the normal pancreas (Supplementary Table S3); 53 of these genes were preferentially expressed in specific tumor cell types (Table 1; gene names are in Supplementary Table S1_2E). In total, 1193 mouse PDA proteins were predicted to be secreted in late-stage KIC tumors (Supplementary Table S4); 209 genes were preferentially expressed in specific tumor cell types (Table 1; gene names are in Supplementary Table S1_2F), but their plasma levels are yet to be determined. 

The tumor-specific markers among the proteins identified by MS from human PDA cell lines and mouse KIC tumors had few differentially expressed proteins in common, but their general patterns of expression were similar. Most of these proteins were differentially expressed in cancer cells, as well as fibroblasts and/or macrophages in KIC tumors, but those that also had high expression levels in the normal pancreas were set aside because they would probably yield a high rate of false positives in screenings for PDA in the patient samples. In summary, MS of the mouse and human PDA samples identified 50 markers in early epithelial neoplasia (Table 1) and a total of 391 non-redundant candidate markers expressed at various stages of cancer progression, from early epithelial cancer to late epithelial cancer and/or mesenchymal cancer or stromal cells in early- and late-stage tumors. 

#### 2.4.3. Approach (3): Pathways of PDA Initiation and Progression 

##### Ras Pathway Genes 

Oncogenic Kras mutations are acquired early in PDA progression, but alone, these mutations only destabilize acinar and ductal cell identity [20]. Additional mutations in tumor suppressor genes and chromatin-modifying genes are required for transition into PDA [2]. To assess the expression of other genes important to Ras activity, we surveyed the cell-type expression of Ras pathway genes (https://www.genome.jp/kegg/; accessed on 22 March 2021) [19]. A total of 197 genes were preferentially expressed in specific cell types in PDA tumors but none were uniquely expressed and only eight were expressed in the early epithelium (Table 1; gene names are in Supplementary Table S1_3G). Thus, the Ras pathway primarily comprises markers identifying later stages of PDA. 

##### Mutated Genes 

We screened genes associated with genetic alterations in human and mouse PDA in scRNAseq from KIC tumors and a normal pancreas to identify potential diagnostic and stage-specific PDA markers. Numerous genetic mutations are associated with PDA in humans, including missense, gene amplification, and homozygous deletion (as summarized on cBioPortal.org) [21,22,23,24]. Missense and gene amplification events are most frequently found in oncogenes and/or dominant negative mutations in tumor suppressor genes. Homozygous deletions typically occur in tumor suppressor genes. Genetic drivers of PDA were further explored in Sleeping Beauty mutagenesis screenings promoting or inhibiting Kras-dependent transformation in KC mice [25] (Table 1; gene names are in Supplementary Table S1_3H–3K).

We also queried the cell-type expression of mutated cancer gene lists commercially available from Tempus and Foundation Medicine (see company websites). The mutation screening kits from these companies were used to identify new mutations not previously observed in PDA. Of the 688 genes listed, 191 genes were differentially expressed in the PDA cells from the KIC mice (117 genes had not been previously characterized by us) (Table 1; animation time course of expression is in Supplementary Figure S6). 

From these five sources of genetic markers, 254 genes were preferentially expressed in the PDA cancer cells and 50 of these were expressed in the early epithelium, with 13 exclusively so (Table 1; gene names and cell-type expression are in Supplementary Table S1_3H–3L). 

##### KEGG Cancer Genes 

The KEGG cancer gene set (530 genes) had 66 genes preferentially expressed in the PDA cancer cells, but only one was expressed in early epithelial cancer cells, while 18 genes were preferentially expressed in stromal cell types (fibroblasts, macrophages and/or lymphocytes) (Table 1; gene names and cell-type expression are in Supplementary Table S1_3M). 

##### Pancreatitis Models 

Healthy acinar cells resist Kras-dependent PDA initiation and progression [26]. Caerulein stimulates intense calcium signaling evoked by the Gq-coupled CCK receptor [27]. Acute hyperstimulation by caerulein causes the dedifferentiation of acinar cells and provokes an innate immune response initiated by dedifferentiated acinar cells [3,12,18]. In otherwise healthy mice, acinar cells re-differentiate and recover normal activity in about seven days, dependent on the activity of two core differentiation transcription factors (dTFs), Ptf1a and Nr5a2 [13,14,18,19,28]. These dTFs are required for normal embryonic acinar cell development and differentiation, as well as acinar cell maintenance in the pancreata of adult patients [29]. These dTFs oppose Kras-mediated acinar cell transformation in PDA [18,19,30]. However, dTF mRNA and protein expression are transiently expunged during acinar cell dedifferentiation provoked by caerulein, providing an opportunity for oncogenic Kras to drive initial steps towards PDA in caerulein-treated mice.

- Calcium pathway genes: Among 1750 genes associated with calcium signaling, 138 were preferentially expressed in specific tumor cell types (Table 1; gene names and cell type expression are in Supplementary Table S1_3N).
- Caerulein: To identify PDA markers that might be differentially expressed in pancreatitis, all candidate PDA marker genes identified above were screened for differential expression in the pancreas of normal mice treated with caerulein; 72 genes were up regulated by the caerulein treatment and 4 genes were down regulated in specific tumor cell types (Table 1; gene names and cell type expression are in Supplementary Table S1_3O and ST1_3P, respectively).
- Differentiation Transcription Factors (dTFs): A total of 154 and 88 genes that were differentially expressed in KIC tumor cells were also elevated or reduced two-fold, respectively, compared to a normal pancreas in dTF KO mice with the acinar-cell-specific deletion of either Ptf1a or Nr5a2 (Table 1; gene names and cell type expression are in Supplementary Table S1_3Q and ST1_3R, respectively).

##### Innate Immune Genes

Finally, because acinar-to-ductal metaplasia (ADM) [31] initiates an AP-1- and NFkB-dependent innate immune response [18], we screened the innate immune and NFkB signaling pathways and found 46 genes that were differentially expressed in specific KIC tumor cells compared to in a normal pancreas (Table 1; gene names and cell type expression are in Supplementary Table S1_3S). 

Some genes first induced in mouse models of pancreatitis were later expressed in early epithelial cancer cells, and some persisted in mesenchymal cells. These are excellent candidate genes to evaluate as early markers in patients at high risk for progression to PDA. The genes we report partially overlap with early marker genes independently identified by analyzing changes in chromatin accessibility and scRNAseq expression in epithelial cells isolated 48 h post-caerulein treatment in KC mice [32]. 

#### 2.4.4. Approach (4): Prognostic Markers 

Many of the genes that were overexpressed in the epithelial and/or mesenchymal cells of the late-stage KIC tumors (such as Dsg2, Sfn, Mal2, Jup, Tspan, Hmga1 and Cd44; Figure 2) were associated with a poor survival rate in patients (Cox coefficient > 0.250; oncolnc.org). For example, members of the ephrin and Eph receptor gene families with the highest expression in late-stage cancer cells were also the most associated with poor survival, whereas genes with little or no expression in the KIC tumors had little impact on survival (Supplementary Figure S7). Efnb2 is particularly noteworthy, as genetic and pharmacologic studies show it plays a role in cancer cell motility and disease progression [33,34,35,36]. Therefore, we queried the TCGA database of genes associated with worse or better survival in pancreatic ductal adenocarcinoma as a potential resource of both tumor-specific prognostic markers and therapeutic targets (oncolnc.org) [37]. 

**Figure 3:**
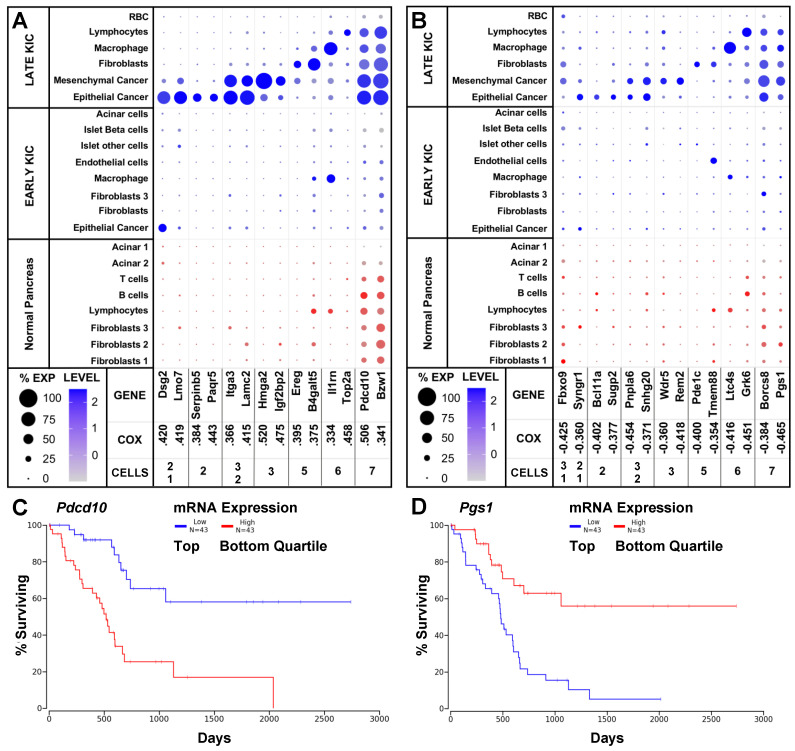
Quantitative dot plots (QDPs) of cell-type distribution and levels of exemplary prognostic PDA markers in mice or human PDA cell lines. PDA markers identified by MS in mouse KIC tumors or conditioned media from human PDA cell lines were associated with the following: (**A**) worse survival prognosis (Cox score ≥ 0.250); or (**B**) better survival prognosis (Cox score ≤ −0.250) in PAAD (oncolnc.org). Candidate genes were further characterized by QDP analysis of scRNAseq from PDA tumors in KIC mice at 60 days (Late KIC) or 40 days (Early KIC) or normal pancreas at 60 days [8]. Representative differentially expressed genes in KIC tumor cell types compared to normal pancreas were detected in the following: (1) Epithelial cancer cells in early-stage KIC mice; (2) Epithelial cancer cells in late-stage KIC mice; (3) Mesenchymal cancer cells in late-stage KIC; (5) Fibroblast classes 1, 2 and/or 3 (F1, F2 or F3) in early- and/or late-stage KIC; and (6) Hematopoietic cells (macrophage, T cells, B cells or lymphocytes). (7) Markers were preferentially expressed in many cell types in KIC tumors compared to normal pancreas. The percentage (%) of cells within a given cell type is represented by the size of the dot; an increased color intensity is correlated with a higher level of expression (blue dots, tumor cell types; red dots, normal pancreas cell types). (**C**,**D**) Cox coefficients are a measure of the association between mRNA expression level and patient survival outcome. Kaplan–Meier curves of patient survival associated with top and bottom quartiles of mRNA expression of (**C**) *Pdcd10* and (**D**) *Pgs1*.

To systematically screen for differentially expressed prognostic markers in PDA, we screened all genes associated with either worse or better survival in human PDA patients (PAAD) for cell-type expression in scRNAseq from KIC and normal mice (representative genes are in Figure 3A,B). We chose a cut-off for Cox coefficient scores greater than 0.250 (1307 genes) or less than −0.250 (2080 genes) because the Kaplan–Meier survival curves of the high and low quartiles began to separate shortly after enrollment, and their separation continued to widen after one and two years (as in Figure 3C,D). 

We found 698 and 372 of the genes differentially expressed in the PDA tumors the in early- and late-KIC mice were associated with worse or better prognoses. The cell-type expression of the differentially expressed genes that were identified among the 500 most highly correlated with worse or better survival is reported (Supplementary Table S1_4U and 4W, respectively). The genes associated with worse survival tended to be more highly expressed in cancer cells, whereas the genes associated with better survival tended to have a higher expression in stomal hematopoietic cells. The top and bottom quartiles of *Pdcd10* (Cox 0.506) and *Pgs1* (Cox −0.465) were associated with dramatically different survival outcomes (Figure 3C,D). Even accounting for the eight long-lived patients with tumors having neuroendocrine features and one control sample in the PAAD dataset, nearly 60% of the patients with low *Pdcd10* or high *Pgs1* expression survived 8 years past diagnosis. Differentially expressed genes with exceptionally high or low Cox coefficient scores may be excellent therapeutic targets. 

### 2.5. Immunohistochemistry Validation of PDA Markers

**Figure 4:**
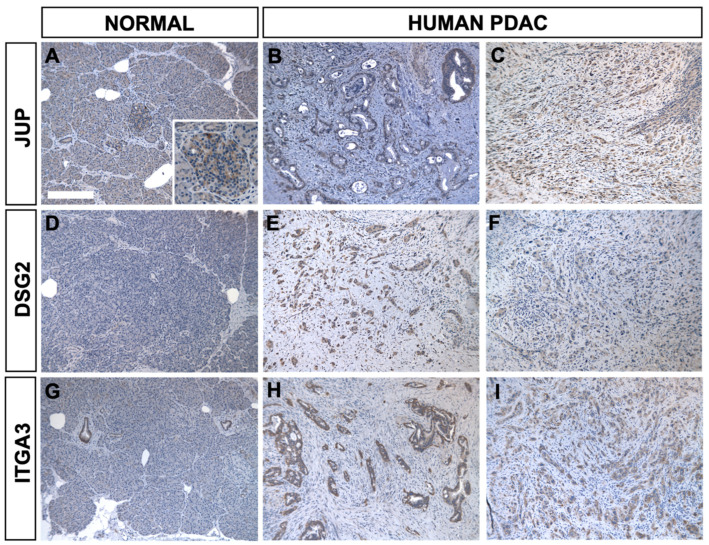
Immunohistochemistry validation of PDA markers in human tumors. Protein expression of JUP, DSG2 and ITGA3 in human normal pancreas (**A**,**D**,**G**) and PDA tumors (**B**,**C**,**E**,**F**,**H**,**I**) corresponded to scRNAseq expression in mouse normal pancreas and KIC tumor (Panel **A**, Scale bar is 250 μm; panels **A**–**I** are 10× magnification). Pancreatic islets express JUP protein (Panel **A**, inset) and mRNA (see Figure 3). Human normal tissue and PDA tumor samples provided by the UT Southwestern Cancer Center patient procurement lab.

Three exemplary markers identified by mass spectrometry (JUP, DSG2 and ITGA3) and shown to be differentially expressed by scRNAseq from KIC tumors were assayed by immunohistochemistry for their tumor-specific expression in paraffin sections of normal and PDA human (Figure 4) and mice samples (Supplementary Figure S8). Supplementary Table S5A,B list the commercially available antibodies for the candidate markers identified by mass spectrometry in human PDA cell lines and mouse tumors, respectively. The tissue expression patterns of protein assayed by IHC and scRNAseq QDP were internally consistent for each marker tested. Thus, scRNAseq provides a convenient and reliable tool for identifying potential diagnostic and prognostic markers. 

### 2.6. Pathway Analysis

Our KEGG and Wiki pathway analysis indicated the differentially expressed genes identified in the mouse scRNAseq samples from the KIC mice were most closely related to human pancreatic, colorectal, hepatocellular and bladder cancers (Supplementary Figure S9). An Enrichr [38] pathway analysis of 1183 genes identified clusters of differentially expressed genes in several interesting pathways, including epithelial–mesenchymal transition (EMT), PI3K-Akt signaling, hyaluronin, and Toll-like receptor signaling. A Cytoscape pathway analysis of differentially expressed genes from the unsupervised screening identified gene clusters predominately in receptor activity, cell–cell communication, sulfur compound binding and processes involved in cell motility (Supplementary Figure S10). 

## 3. Discussion

Multiple cell-type and stage-specific markers were identified from each of the candidate gene searches for markers preferentially expressed in mouse models of pancreatitis, acinar-to-ductal metaplasia, tumors in KC and KIC mice, or human PDA cancer cell lines (summarized in Table 1). Many genes act in multiple pathways. In total, 2165 non-redundant genes differentially expressed in PDA were identified. 

Early markers first expressed in epithelial cancer cells in early-KIC mice were preferentially enriched in the caerulein-treated mice (Caer) and the pancreas-specific knockouts of the two differentiation transcription factors, Ptf1a and Nr5a2 (ko DTF Up/Down). Later stage markers were enriched in genes from pathways known to be involved in PDA initiation and progression and in an unsupervised screening of preferential gene expression in early- vs. late-stage PDA tumors in KIC mice. Another rich source of late-stage markers was identified among the top 1307 genes associated with worse survival in PDA patients (Cox ≥ 0.250, oncolnc PAAD). 

The etiology of cancer initiation and progression is difficult to investigate in patients but can be characterized in mouse models of pancreatitis and PDA. We identified candidate diagnostic and prognostic markers expressed in mouse PDA cancer and stromal cells at specific stages of pancreatitis and PDA initiation and progression. Cell-type specific diagnostic markers can be assessed in blood, other body fluids and tissue specimens to validate their utility in identifying early- and later-stage PDA cancer in patients. Some of these proteins are secreted, while others might be concentrated in exosomes, and their mRNA may also be detected in exosomes and/or platelets [38]. The markers that are expressed in mouse models of pancreatitis may be useful for identifying PDA progression in high-risk patients.

Other stage-specific markers are associated with either longer or shorter survival upon diagnosis of PDA and may be excellent targets for stage-specific treatments. An analysis of the cell-type expression of the 1307 genes whose elevated expressions are most highly correlated with poor survival in PAAD (Cox ≥ 0.250, oncolnc.org) yielded 698 (53%) differentially expressed genes, primarily in late-stage cancer cells. By contrast, the 2080 genes associated with better survival in PDA yielded 369 (18%) differentially expressed genes, mostly in stromal cell types. Many of these genes were previously implicated in cancer. For example, *EFNB2*, *EPHA2* and *ITGA3*, all associated with worse prognoses, are co-expressed in cell motility pathways, and all were highly expressed in late-stage KIC epithelial and mesenchymal cancer (Figure 3A and Figure 4, and Supplementary Figure S7). Genetic and pharmacologic studies indicate *EFNB2* plays a role in cell migration but not cell survival [31,32,33,34]. *ITGA3* encodes integrin alpha-3, which binds extracellular matrix proteins, and its elevated expression is implicated in metastatic cancer [39]. The adhesion receptor Gpr56 (*ADGR1*) was identified by MS to be expressed in two human PDA cell lines, AsPC-1 and PL-45, but not BxPC-1, MIA-PaCa-2 or PANC-1, consistent with earlier findings of low protein expression in later cell lines [40]. *Gpr56* is co-expressed with *Itga3* in late-stage KIC mesenchymal cancer cells, and each interacts with EGFR pathway components that promote PDA (string-db.org) [41,42,43]. The differentially expressed genes in the interactome are listed in Supplementary Table S1_3T. 

In summary, we demonstrate the utility of evaluating quantitative dot plots of scRNAseq data from early- and late-stage KIC mice to identify marker gene expression for specific cell types and stages of PDA progression. Using this approach, we characterized 2165 individual genes expressed in various cell types in multiple mouse models of pancreatitis, as well as early- and late-stage PDA, compared to those in a normal pancreas (Supplementary Table S1). The differentially expressed genes are candidates for stage-specific diagnostic markers for patients at risk of progression to PDA, for the staging of PDA patients and for evaluating disease recurrence after surgical resection of PDA tumors. Furthermore, we identified 1067 differentially expressed genes that are associated with either worse (698 genes) or better (369 genes) survival rates for PDA (Cox coefficient ≥ 0.250 or ≤ −0.250, respectively; oncolnc.org). The characterization of these candidate markers in patient samples from clinics throughout the world will help identify definitive sets of stage-specific diagnostic and prognostic markers and constitutes an initial step towards identifying improved therapeutics. 

## 4. Materials and Methods

Mass Spectrometry. Quantitative mass spectrometric LC-MS/MS analysis was performed as described [9].

Cell Lines. Human pancreatic cancer and normal duct cell lines were obtained from ATCC (https://www.atcc.org accessed on 1 December 2012; all cell lines accessed before 2013). PANC-1 (CRL-1469); MIA-PaCa-2 (CRM-CRL-1420); ASPC-1 (CRL-1682); PL-45 (CRL-2558); BxPc-3 (CRL-1687); and hTERT-HPNE (CRL-4023).

Mice. KIC, pancreas-specific expression of Kras^G12D^ and deletion of Cdkn2a (LSL_Kras^G12D/+^; Cdnk2a^f/f^; Ptf1a::Cre) [7]. KC, pancreas-specific expression of Kras^G12D^ (LSL_Kras^G12D/+^; Ptf1a::Cre) [7]. Pancreas-specific Ptf1a and Nr5a2 KO [13,19].

Caerulein treatment. Normal mice (12 weeks of age) were treated with caerulein as described [19].

Comparison of RNA and secreted protein expression in human PDA cell lines. Relative levels of RNAseq (Log FPKM; obtained from CCLE, Broad Institute) and secreted protein in conditioned media (quantitative MS; [8,9]) were compared for the cell lines AsPC-1, BxPc-3, MIA-PaCa-2 and PANC-1. Refer to Supplementary Figure S4 for details.

Dot plots and heatmaps. Quantitative dot plots (QDPs) were generated using Seurat v4.0 R package. R version 4.0.3 was used for all computations.

Immunohistochemistry. Paraffin-embedded human PDA tissue sections on glass slides were obtained from the Harold C. Simmons Comprehensive Cancer Center Tissue Management Shared Resource. Mouse PDA samples were from KIC mice [7]. Sections were incubated at 60 °C for 30 min then quickly dipped in ClearRight3 (Richard-Allan Scientific 22-046341; San Diego, CA, USA) for 20 min to deparaffinate. Sections were then rehydrated by dipping in decreasing concentrations of ethanol followed by final 15 min bath in dH_2_O. Antigen retrieval was performed by incubation in Antigen Unmasking Solution (Vector Labs H3300; Newark, CA, USA) for 10 min followed by high temperature/pressure retrieval (Aptum Biologics RR2100-EU 2100-Retriever; Southampton, UK) for 60 min. Sections were cooled and equilibrated in dH_2_O for 10 min. Endogenous hydrogen peroxide activity was quenched by incubation in 3% H_2_O_2_ for 30 min followed by 10 min rinse in dH_2_O. Background binding issues were blocked using 5% normal goat serum (Cell Signaling Technology #5425; Danvers, MA, USA) in PBS + 0.05% Tween 20 (PBS-T) for 60 min at RT. Primary antibodies were diluted in 2.5% normal goat serum in PBS-T and incubated on tissue sections O/N at 4 °C in a humid chamber. Sections were washed in PBS-T for 15 min. Sections were incubated with HRP-conjugated secondary antibodies for 60 min RT, then washed in PBS-T for 14 min. Sections were equilibrated in DAB Buffer (DBA Substrate Kit, Thermoscientific #34002; Waltham, MA, USA) for 10 min RT followed by incubation for varying times in 1× DAB substrate in supplied buffer. Reactions were stopped by washing in PBS-T for 15 min. Counterstaining was performed by briefly dipping the sections in Harris hematoxylin (Sigma-Aldrich HHS32; St Louis, MO, USA) followed by extensive washing in warm tap H_2_O. Coverslips were affixed using Permount Mounting Medium (Fisher SP15-100; Hampton, NH, USA). Images were acquired using a Leica DMRXE microscope (Wetzlar, Germany). Minimal optimization of images was performed using Preview version 8.1.

Primary antibodies were rabbit anti-desmoglein2 (Novus Biologicals NBP1-33374; dilution 1:500; Centennial, CO, USA), rabbit anti-junction plakoglobin (Proteintech 11146-1-AP; dilution 1:50; Rosemont, IL, USA) and mouse anti-Integrin alpha 3 (Proteintech 66070-1-Ig; dilution 1:4000; Rosemont, IL, USA). Secondary antibodies were HRP-conjugated Goat Anti-Rabbit IgG Polymer Detection Kit peroxidase (Immpress MP7451; Vector Labs, Newark, CA, USA) and Goat Anti-Mouse IgG Polymer Detection Kit peroxidase (Immpress MP 7452; Vector Labs, Newark, CA, USA).

**Pathway Analysis.** Enrichr [38], an online tool for overenrichment analysis (ORA), was used to identify pathways containing the most genes that were differentially expressed in PDA tumors in KIC mice. The set of 1187 markers was queried against four databases (KEGG, WikiPathways, Reactome and MSigDB Hallmarks [44,45,46,47]) to identify biologically relevant pathways. The combined score assessed the magnitude of pathway enrichment considering the ratio of genes in both the query list of 1187 markers and the pathway gene set, the number of genes in the pathway and the statistical significance of inclusion (adjusted *p*-value < 0.002). Pathways with the highest combined score were plotted using DOSE [48] and ggplot2 packages in R. The color of each dot represents the adjusted *p* value while the size is proportional to the combined score (magnitude of pathway enrichment in gene list calculated in Enrichr) (see Supplementary Figure S9).

Pathway analysis of the 503 differentially expressed genes identified by an unsupervised comparison of scRNAseq levels in normal pancreas vs. early- and late-stage KIC queried multiple databases (GO:CC—GO cellular component; GO:MF—GO molecular function; GO:BP—GO biological process; KEGG—KEGG pathways; REAC—Reactome pathways; TF—Regulatory motifs for these genes; HPA—Human protein atlas; CORUM—Comprehensive resource of mammalian protein complexes; and HP—Human phenotype ontology (see Supplementary Figures S1–S10, Supplementary Tables S1–S5).

## Figures and Tables

**Table 1:** Cell-type expression of differentially expressed genes in KIC neoplasia.

Supplementary Table S1	Query ^a^	Total Genes #	KIC Cell Type Classification								
			E.epi	EL.epi	EL.epi + mes	L.epi	L.e + m	L.mes	EL.fib	EL.h	EL.all
			1	1 + 2	1 + 2 + 3	2	2 + 3	3	5	6	7
ST1_1C	NvEvL unsup	503	7	34	9	12	98	56	7	47	233
ST1_2D	mMS	53	2	4	0	5	5	2	5	30	nd
ST1_2E	msMS	209	4	9	5	37	61	10	10	73	nd
ST1_2F	hMS	187	4	15	7	34	57	17	16	37	nd
ST1_3G	Ras Path	197	0	7	3	25	65	18	18	62	nd
ST1_3H	Mutant cBP	16	0	2	0	3	7	4	0	0	nd
ST1_3I	AMP cBP	13	0	4	1	0	4	4	0	0	nd
ST1_3J	Del cBP	17	1	4	1	1	4	6	0	0	nd
ST1_3K	SB	17	0	1	2	4	9	0	0	1	nd
ST1_3L	Tempus + FM	191	12	6	16	47	50	23	9	28	nd
ST1_3M	KEGG cancer	66	0	0	1	5	33	9	4	14	nd
ST1_3N	Ca^2+^ Path	138	1	5	3	24	54	19	8	24	nd
ST1_3O	Caer Up	72	1	7	3	9	26	2	5	15	nd
ST1_3P	Caer Down	4	0	1	0	0	1	1	0	1	nd
ST1_3Q	ko dTF Up	154	5	20	6	25	40	15	5	38	nd
ST1_3R	ko dTF Down	88	4	6	5	12	23	10	8	20	nd
ST1_3S	Innate Imm	46	2	1	0	8	3	5	5	22	nd
ST1_3T	ITGA3-Gpr56	128	1	5	1	20	54	14	14	19	nd
ST1_4U	Cox Hi_500	202	0	4	2	21	53	6	3	11	102
ST1_4W	Cox Lo_500	191	0	3	0	13	21	3	4	7	140
ST1_Y	Panc CT_ID	41	0	1	2	0	0	0	4	12	22

Candidate gene signature type identified in early- and/or late-stage KIC scRNAseq samples. Total gene # differentially expressed in PDA. **^a^** see Supplementary Table S1 for the list of gene names in each search query. *nd*, not determined. KIC cell-type classification defined in Figure 1 and Supplementary Figure S1: E: early-stage KIC. L: late-stage KIC. E.epi (cell type 1): early KIC epithelial cancer. EL.epi (cell types 1 + 2): early- and late-stage KIC epithelial cancer. EL.epi + mes (cell types 1 + 2 + 3): early- and late-stage KIC epithelial and late-stage mesenchymal cancer. L.epi (cell type 2): late-stage KIC epithelial cancer. L.epi + mes (L.e+m; cell types 2 + 3): late-stage KIC epithelial and mesenchymal cancer. L.mes (cell type 3): late-stage KIC mesenchymal cancer. EL.fib (cell type 5): early- and/or late-stage KIC tumor fibroblasts. EL.h (cell type 6): early- and/or late-stage KIC tumor hematopoietic cells (macrophages or lymphocytes). EL.all (7): genes broadly expressed in early- and/or late-stage KIC cancer and stromal cell types 1–6. **^a^ Gene signature type**: **NvEvL unsup**, unsupervised screening of scRNAseq of normal pancreas vs. early-stage KIC and late-stage KIC; **mMS**, mouse KIC PDA mass spec; **msMS**, mouse KIC PDA mass spec predicted secreted proteins; **hMS**, mass spec of conditioned media from human PDA cell lines; **Ras Path**, non-redundant genes in KEGG pathways containing Kras; **Mutant cBP**, missense mutations in cancer cBioPortal (top 25); **AMP cBP**, gene amplification in cancer cBioPortal (top 25); **Del cBP**, gene deletions in cancer cBioPortal (top 25); **SB**, Sleeping Beauty mutagenesis in mouse PDA (top 25); **Tempus + FM**, Tempus and Foundation Medicine mutant genes in cancer; **KEGG cancer**, mutant genes in cancer pathways; **Ca^2+^ Path**, calcium signaling pathway genes; **Caer Up**, elevated differential gene expression in pancreas of normal mice treated with caerulein; **Caer Down**, decreased differential gene expression in pancreas of normal mice treated with caerulein; **ko dTF Up**, elevated differential gene expression in pancreas of transcription factor (dTF, *Ptf1a* or *Nr5a2*) knockouts; **ko dTF Down**, decreased differential gene expression in pancreas of transcription factor (dTF, *Ptf1a* or *Nr5a2*) knockouts; **Innate Imm**, immune and NFkB signaling pathway genes; **ITGA3-Gpr56**, ITGA3 and Gpr56 STRING interactome genes; **Cox Hi**, top 500 genes associated with worse survival in human PDA patients (Cox coefficient, PAAD, oncolnc.org); **Cox Lo**, bottom 500 genes associated with better survival in human PDA patients (Cox coefficient, PAAD, oncolnc.org); **Panc CT_ID**, cell-type identifier genes with cell-type-specific expression in normal mouse pancreas or PDA tumors in KIC mice.

## Data Availability

Data is contained within the article, Supplementary Material, or references cited.

## Supplementary Materials

The following supporting information can be downloaded at: https://www.mdpi.com/article/10.3390/ijms25126619/s1.

## Abbreviations

MS, mass spectrometry; scRNAseq, single cell RNA sequencing; ADM, acinar-to-ductal metaplasia; PDA, pancreatic ductal adenocarcinoma; PAAD, TCGA pancreatic adenocarcinoma; TCGA, the cancer genome atlas; KIC, mouse model of PDA; QDP, quantitative dot plot; dTF, differentiation transcription factors of pancreatic acinar cells (Ptf1a, Nr5a2); T2D, type 2 diabetes; tSNE, t-distributed stochastic neighbor embedding.
