# Supplementary figures and images for: Diagnostic and Prognostic Markers for Pancreatitis and Pancreatic Ductal Adenocarcinoma
Source: Int J Mol Sci. 2024 Jun 16;25(12):6619. doi: 10.3390/ijms25126619 (PMC11204091; doi:10.3390/ijms25126619)

## Slide 1
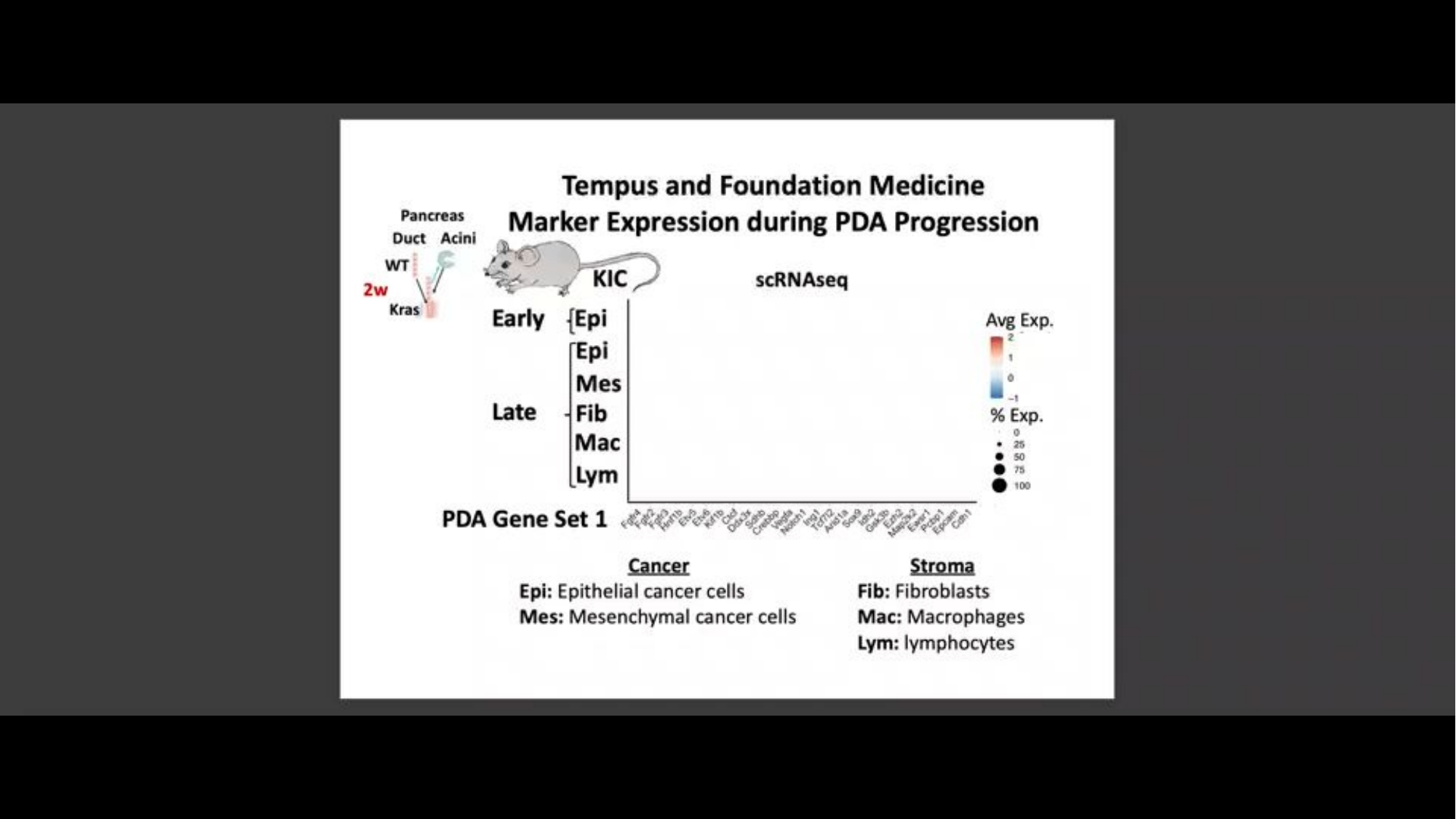

Supplement: Supplementary file 1 [file ijms-25-06619-s001.zip › Figure S6_TempusFM_PDA_animation.pptx]
