# Supplementary material for: Diagnostic and Prognostic Markers for Pancreatitis and Pancreatic Ductal Adenocarcinoma
Source: Int J Mol Sci. 2024 Jun 16;25(12):6619. doi: 10.3390/ijms25126619 (PMC11204091; doi:10.3390/ijms25126619)

scRNAseq Markers 2 _SF1B

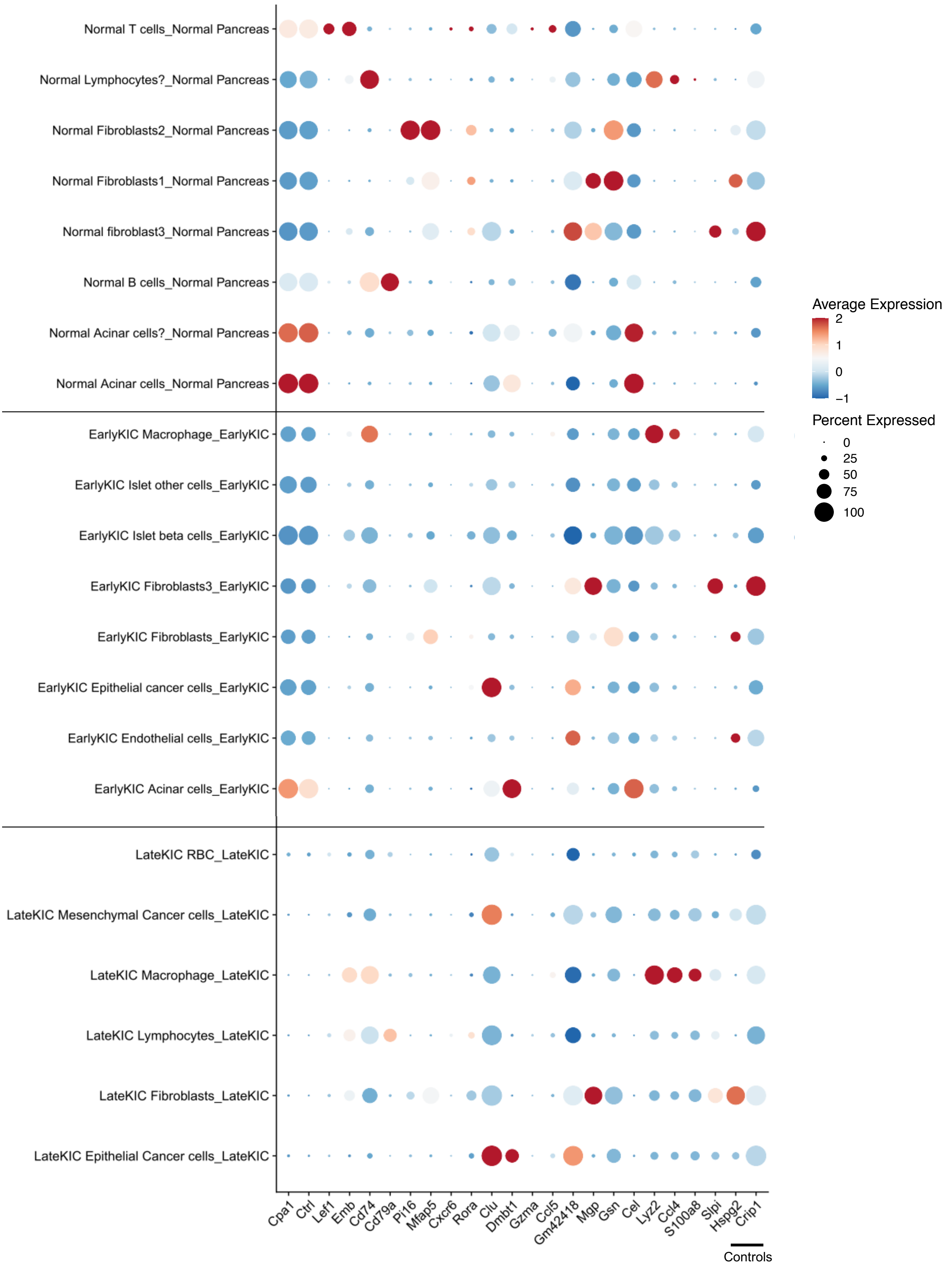

scRNAseq Markers 2 _SF1B

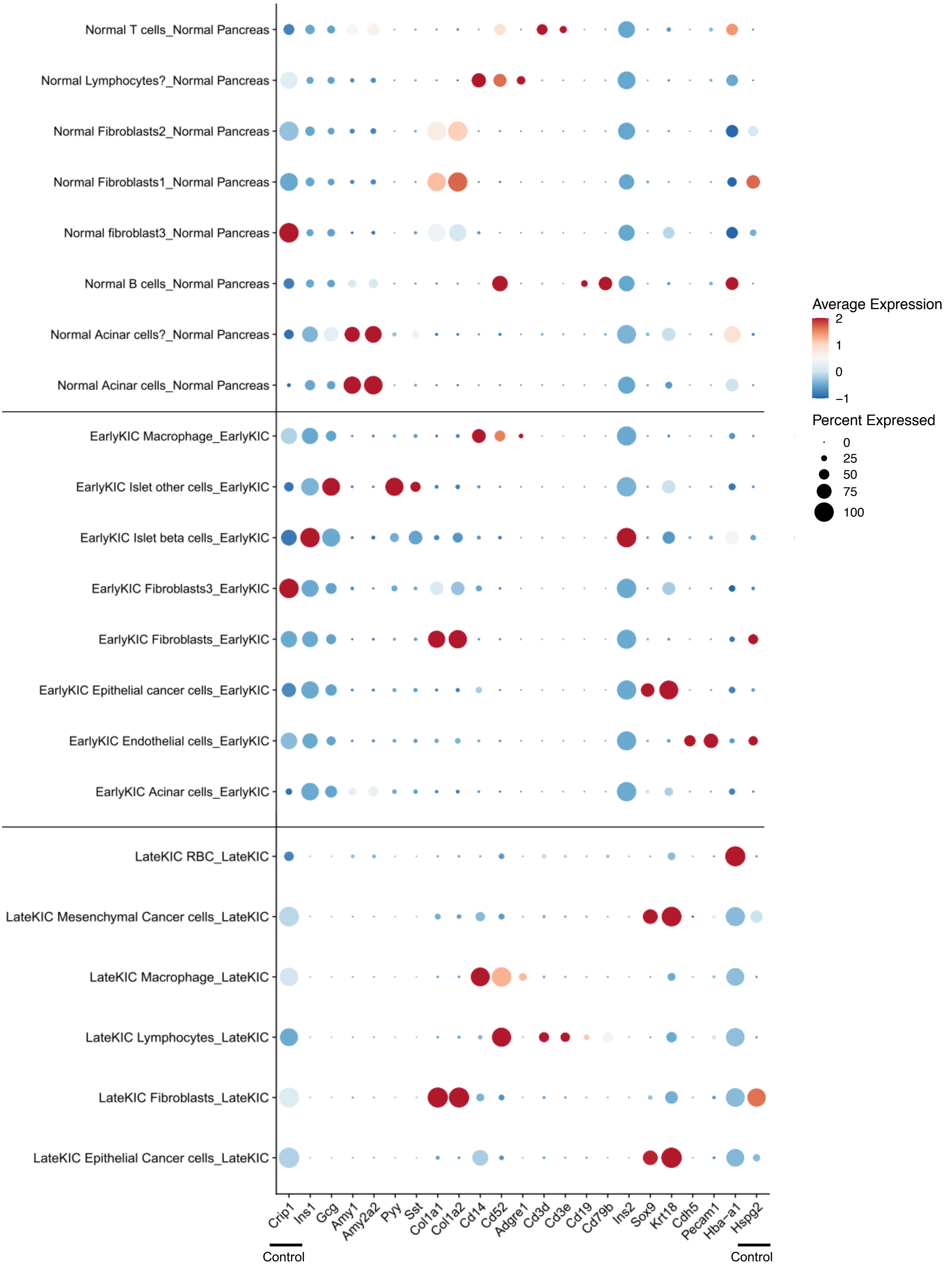

Supplement: Supplementary file 1 [file ijms-25-06619-s001.zip › Figrue S1B_scRNA_control2_PancMark2DP_x4B.pdf.zip!/SF1B_scRNA_control2_PancMark2DP_x4B.pdf]
