# Supplementary material for: Diagnostic and Prognostic Markers for Pancreatitis and Pancreatic Ductal Adenocarcinoma
Source: Int J Mol Sci. 2024 Jun 16;25(12):6619. doi: 10.3390/ijms25126619 (PMC11204091; doi:10.3390/ijms25126619)
Supplement: Supplementary file 1 [file ijms-25-06619-s001.zip › Figure S10_Cytoscape.pdf.zip!/SF10_Cytoscape.pdf]

SF10

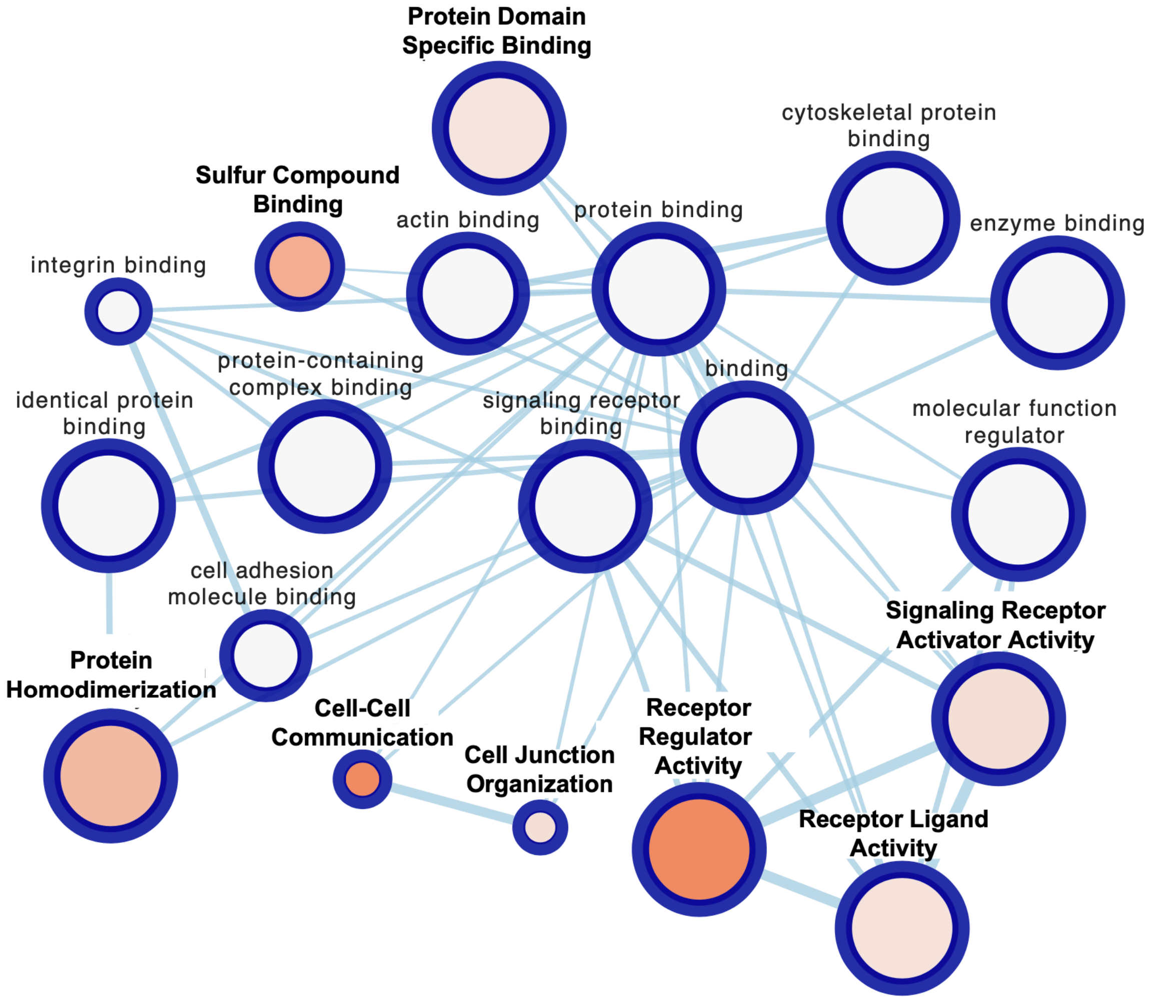
Protein Domain Specific Binding
cytoskeletal protein binding
Sulfur Compound Binding
actin binding
protein binding
enzyme binding
integrin binding
protein-containing complex binding
binding
identical protein binding
signaling receptor binding
molecular function regulator
cell adhesion molecule binding
Signaling Receptor Activator Activity
Protein Homodimerization
Cell-Cell Communication
Cell Junction Organization
Receptor Regulator Activity
Receptor Ligand Activity
