# Supplementary material for: Diagnostic and Prognostic Markers for Pancreatitis and Pancreatic Ductal Adenocarcinoma
Source: Int J Mol Sci. 2024 Jun 16;25(12):6619. doi: 10.3390/ijms25126619 (PMC11204091; doi:10.3390/ijms25126619)
Supplement: Supplementary file 1 [file ijms-25-06619-s001.zip › Figure S1A_scRNAseq_HHcontrols_x4.pdf.zip!/SF1A_scRNAseq_HHcontrols_x4.pdf]

SF1A

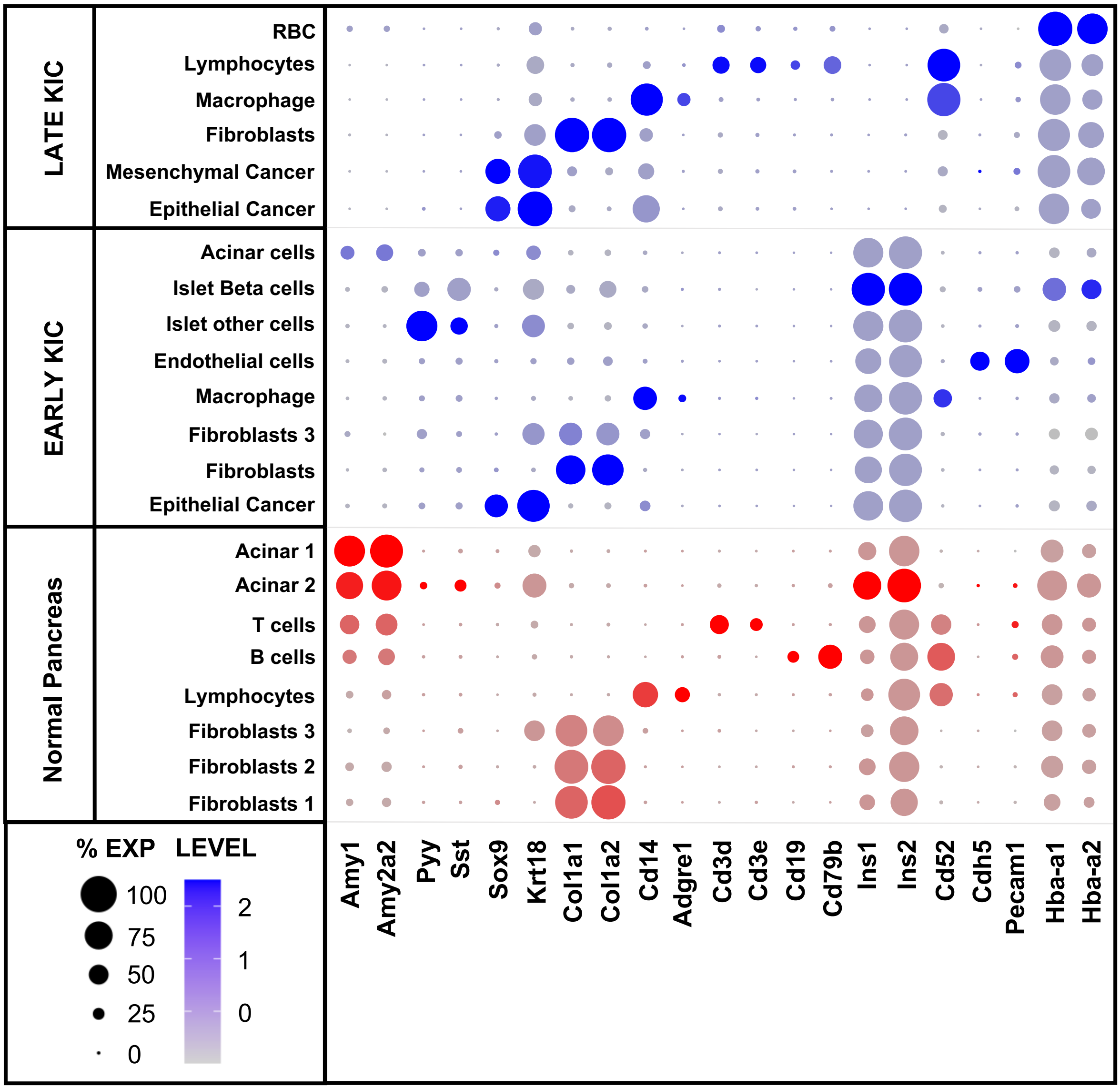

LATE KIC
RBC
Lymphocytes
Macrophage
Fibroblasts
Mesenchymal Cancer
Epithelial Cancer
EARLY KIC
Acinar cells
Islet Beta cells
Islet other cells
Endothelial cells
Macrophage
Fibroblasts 3
Fibroblasts
Epithelial Cancer
Normal Pancreas
Acinar 1
Acinar 2
T cells
B cells
Lymphocytes
Fibroblasts 3
Fibroblasts 2
Fibroblasts 1
% EXP
100
75
50
25
0
LEVEL
2
1
0
Amy1
Amy2a2
Pyy
Sst
Sox9
Krt18
Col1a1
Col1a2
Cd14
Adgre1
Cd3d
Cd3e
Cd19
Cd79b
Ins1
Ins2
Cd52
Cdh5
Pecam1
Hba-a1
Hba-a2
