# Supplementary material for: Diagnostic and Prognostic Markers for Pancreatitis and Pancreatic Ductal Adenocarcinoma
Source: Int J Mol Sci. 2024 Jun 16;25(12):6619. doi: 10.3390/ijms25126619 (PMC11204091; doi:10.3390/ijms25126619)
Supplement: Supplementary file 1 [file ijms-25-06619-s001.zip › Figure S2AB_violinQDPv2_x6AB.pdf.zip!/SF2AB_violinQDPv2_x6AB.pdf]

SF2

A

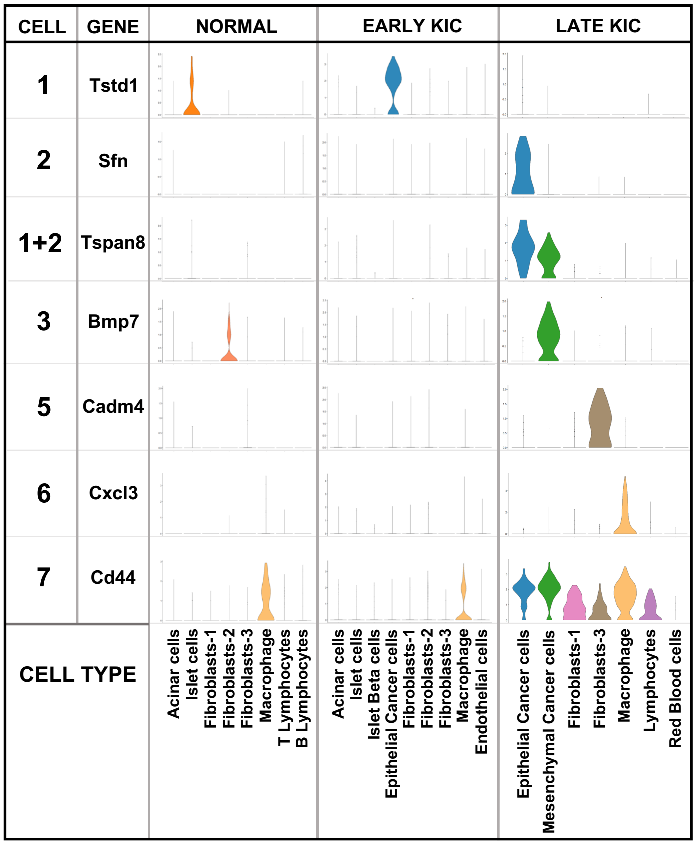
CELL
GENE
NORMAL
EARLY KIC
LATE KIC
1
Tstd1
2
Sfn
1+2
Tspan8
3
Bmp7
5
Cadm4
6
Cxcl3
7
Cd44
CELL TYPE
Acinar cells
Islet cells
Fibroblasts-1
Fibroblasts-2
Fibroblasts-3
Macrophage
T Lymphocytes
B Lymphocytes
Acinar cells
Islet cells
Islet Beta cells
Epithelial Cancer cells
Fibroblasts-1
Fibroblasts-2
Fibroblasts-3
Macrophage
Endothelial cells
Epithelial Cancer cells
Mesenchymal Cancer cells
Fibroblasts-1
Fibroblasts-3
Macrophage
Lymphocytes
Red Blood cells

B

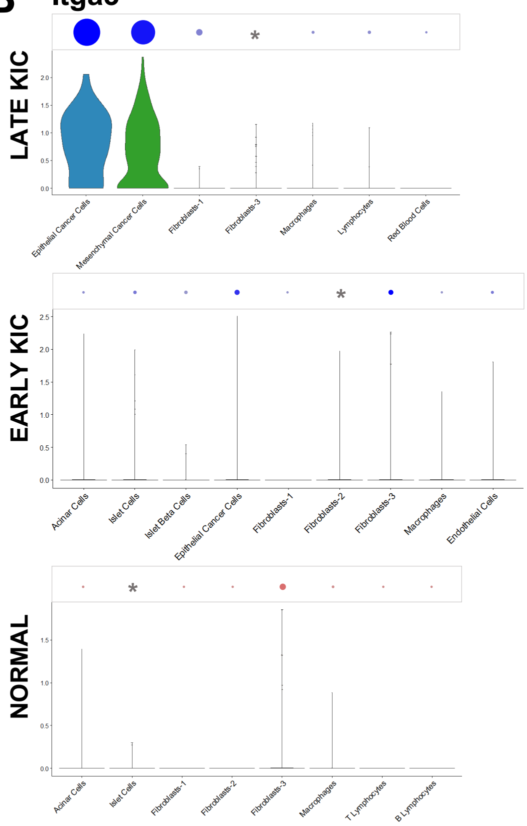
Itga3
LATE KIC
2.0
1.5
1.0
0.5
0.0
Epithelial Cancer Cells
Mesenchymal Cancer Cells
Fibroblasts-1
Fibroblasts-3
Macrophages
Lymphocytes
Red Blood Cells
EARLY KIC
2.5
2.0
1.5
1.0
0.5
0.0
Acinar Cells
Islet Cells
Islet Beta Cells
Epithelial Cancer Cells
Fibroblasts-1
Fibroblasts-2
Fibroblasts-3
Macrophages
Endothelial Cells
NORMAL
1.5
1.0
0.5
0.0
Acinar Cells
Islet Cells
Fibroblasts-1
Fibroblasts-2
Fibroblasts-3
Macrophages
T Lymphocytes
B Lymphocytes
