# Supplementary material for: Diagnostic and Prognostic Markers for Pancreatitis and Pancreatic Ductal Adenocarcinoma
Source: Int J Mol Sci. 2024 Jun 16;25(12):6619. doi: 10.3390/ijms25126619 (PMC11204091; doi:10.3390/ijms25126619)
Supplement: Supplementary file 1 [file ijms-25-06619-s001.zip › Figure S3_HuPDAvennDiagram_x2.pdf.zip!/SF3_HuPDAvennDiagram_x2.pdf]

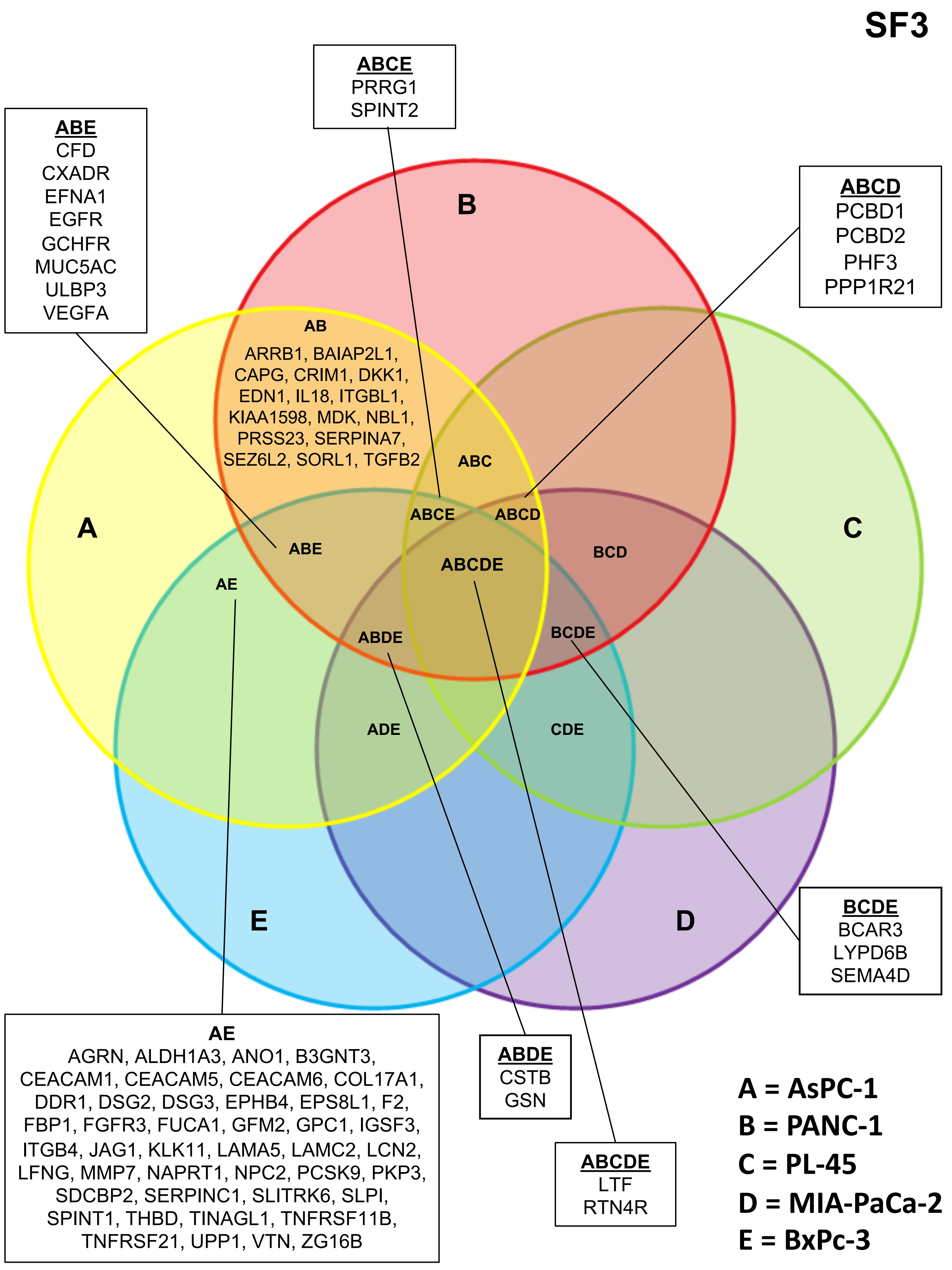
SF3
ABCE
PRRG1
SPINT2
ABE
CFD
CXADR
EFNA1
EGFR
GCHFR
MUC5AC
ULBP3
VEGFA
ABCD
PCBD1
PCBD2
PHF3
PPP1R21
B
AB
ARRB1, BAIAP2L1, CAPG, CRIM1, DKK1, EDN1, IL18, ITGBL1, KIAA1598, MDK, NBL1, PRSS23, SERPINA7, SEZ6L2, SORL1, TGFB2
ABC
ABCE
ABCD
A
C
ABE
ABCDE
BCD
AE
ABDE
BCDE
ADE
CDE
E
D
BCDE
BCAR3
LYPD6B
SEMA4D
AE
AGRN, ALDH1A3, ANO1, B3GNT3, CEACAM1, CEACAM5, CEACAM6, COL17A1, DDR1, DSG2, DSG3, EPHB4, EPS8L1, F2, FBP1, FGFR3, FUCA1, GFM2, GPC1, IGSF3, ITGB4, JAG1, KLK11, LAMA5, LAMC2, LCN2, LFNG, MMP7, NAPRT1, NPC2, PCSK9, PKP3, SDCBP2, SERPINC1, SLITRK6, SLPI, SPINT1, THBD, TINAGL1, TNFRSF11B, TNFRSF21, UPP1, VTN, ZG16B
ABDE
CSTB
GSN
ABCDE
LTF
RTN4R
A = AsPC-1
B = PANC-1
C = PL-45
D = MIA-PaCa-2
E = BxPc-3
