# Supplementary material for: Diagnostic and Prognostic Markers for Pancreatitis and Pancreatic Ductal Adenocarcinoma
Source: Int J Mol Sci. 2024 Jun 16;25(12):6619. doi: 10.3390/ijms25126619 (PMC11204091; doi:10.3390/ijms25126619)

SF4

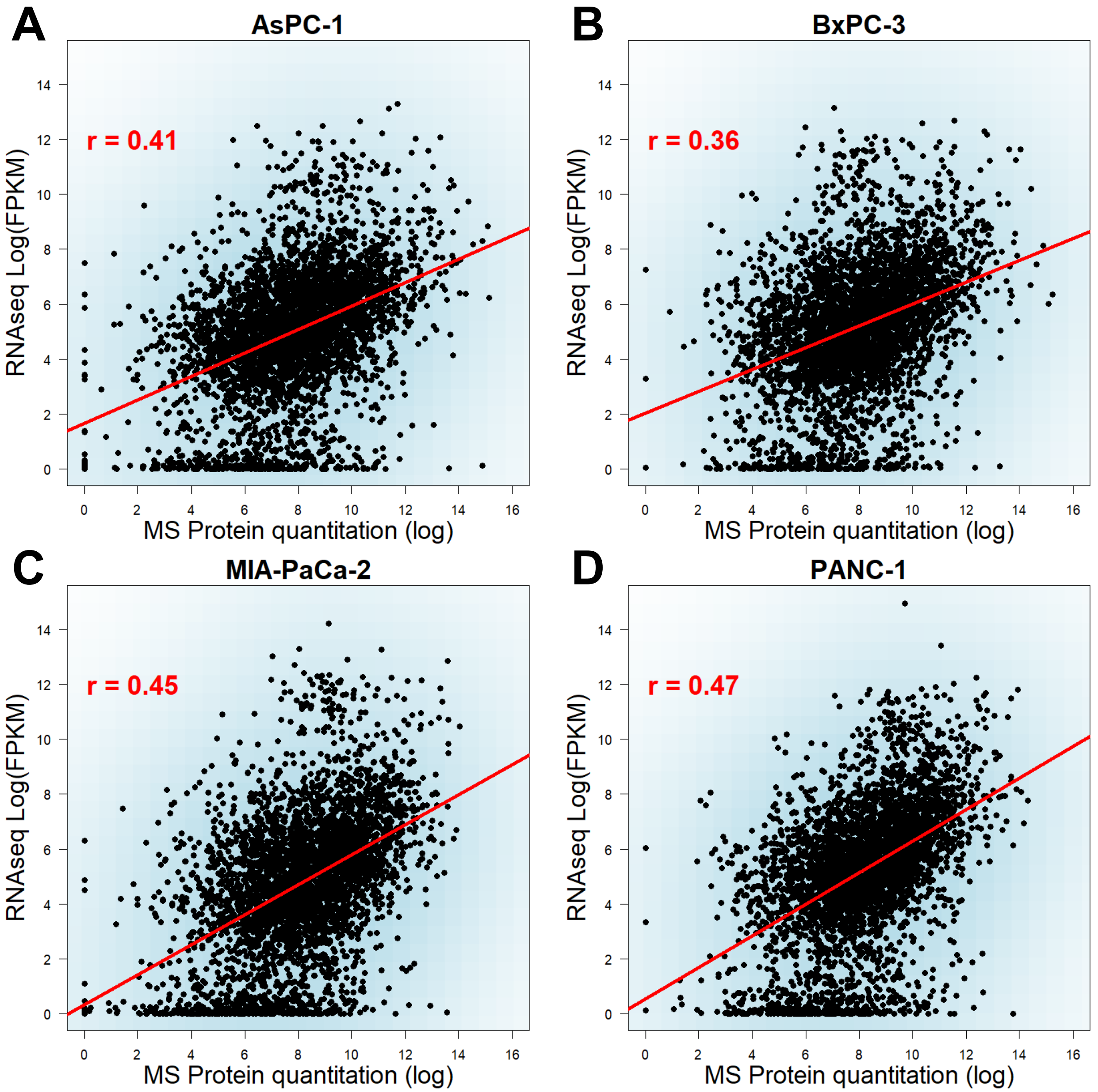


E

| | AsPC-1 | BxPC-3 | Mia PaCa | PANC-1 |
|---|---|---|---|---|
| **Pearson** | 0.411 | 0.355 | 0.453 | 0.468 |
| **Spearman** | 0.416 | 0.371 | 0.458 | 0.490 |

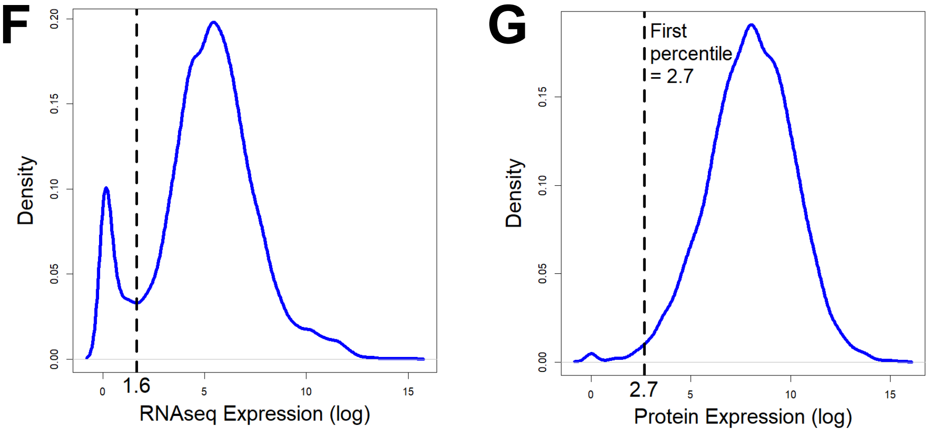

Supplement: Supplementary file 1 [file ijms-25-06619-s001.zip › Figure S4_RNAvProtCellLines_x3.pdf.zip!/SF4_RNAvProtCellLines_x3.pdf]
