# Supplementary material for: Diagnostic and Prognostic Markers for Pancreatitis and Pancreatic Ductal Adenocarcinoma
Source: Int J Mol Sci. 2024 Jun 16;25(12):6619. doi: 10.3390/ijms25126619 (PMC11204091; doi:10.3390/ijms25126619)
Supplement: Supplementary file 1 [file ijms-25-06619-s001.zip › Figure S5_MSquant_KC_KIC_x1.pdf.zip!/SF5_MSquant_KC_KIC_x1.pdf]

SF5

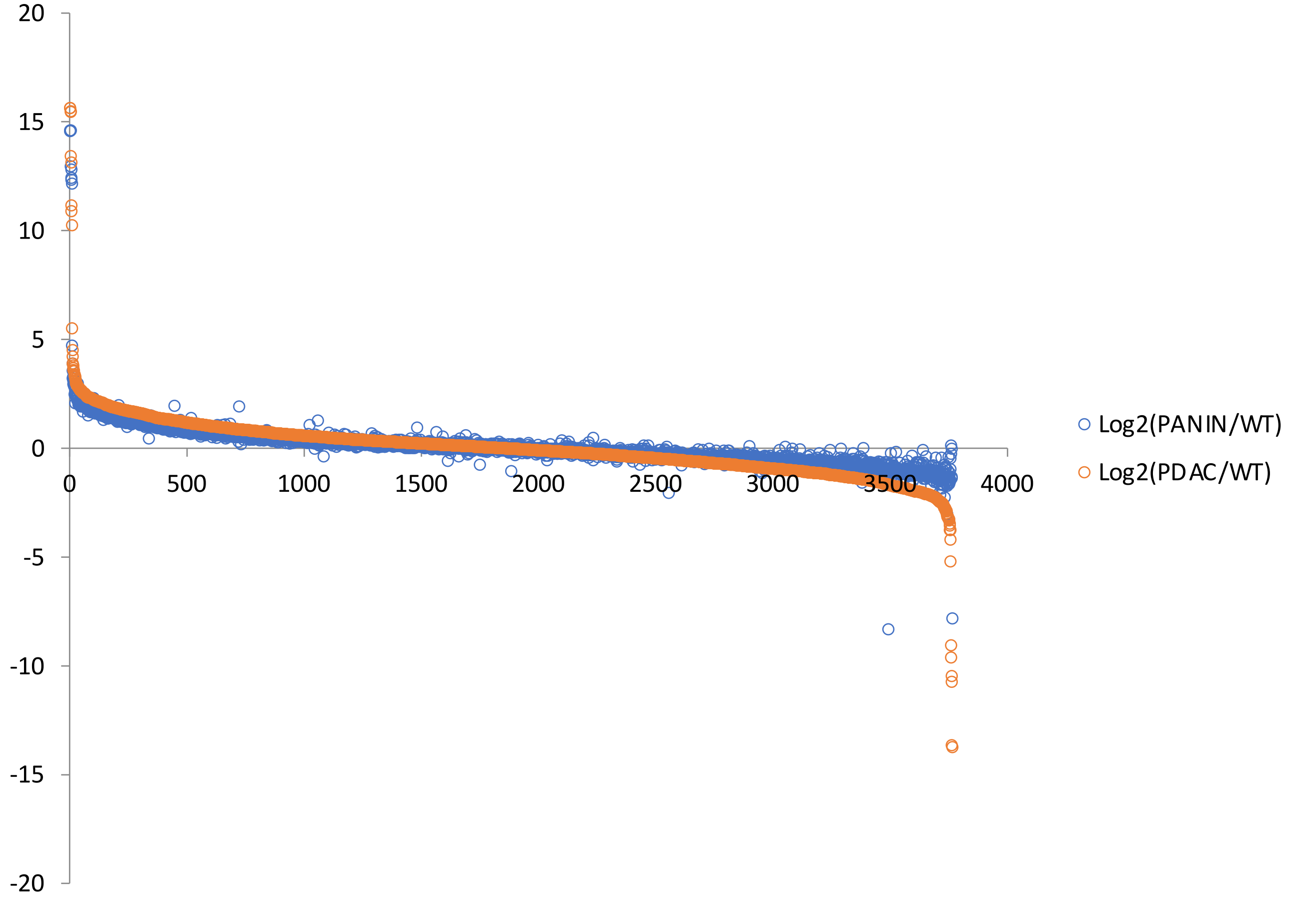

20
15
10
5
0
-5
-10
-15
-20
0
500
1000
1500
2000
2500
3000
3500
4000
Log2(PANIN/WT)
Log2(PDAC/WT)
