# Supplementary material for: Diagnostic and Prognostic Markers for Pancreatitis and Pancreatic Ductal Adenocarcinoma
Source: Int J Mol Sci. 2024 Jun 16;25(12):6619. doi: 10.3390/ijms25126619 (PMC11204091; doi:10.3390/ijms25126619)
Supplement: Supplementary file 1 [file ijms-25-06619-s001.zip › Figure S7_eph FINAL Naviya_PORTRAIT.pdf.zip!/SF7_eph FINAL Naviya_PORTRAIT.pdf]

SF7

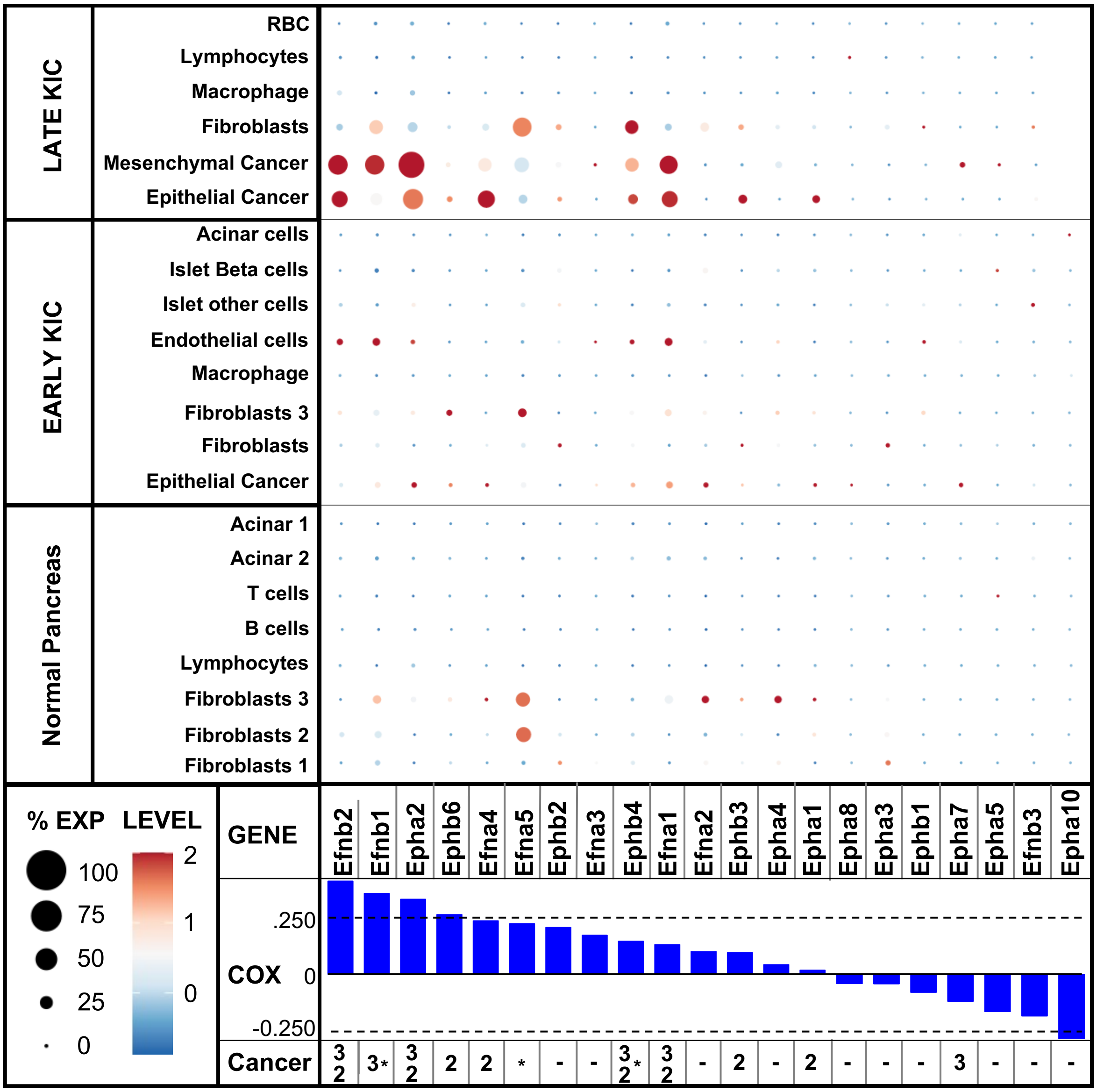

LATE KIC
RBC
Lymphocytes
Macrophage
Fibroblasts
Mesenchymal Cancer
Epithelial Cancer
EARLY KIC
Acinar cells
Islet Beta cells
Islet other cells
Endothelial cells
Macrophage
Fibroblasts 3
Fibroblasts
Epithelial Cancer
Normal Pancreas
Acinar 1
Acinar 2
T cells
B cells
Lymphocytes
Fibroblasts 3
Fibroblasts 2
Fibroblasts 1
% EXP
100
75
50
25
0
LEVEL
2
1
0
GENE
Efnb2
Efnb1
Epha2
Ephb6
Efna4
Efna5
Ephb2
Efna3
Ephb4
Efna1
Efna2
Ephb3
Epha4
Epha1
Epha8
Epha3
Ephb1
Epha7
Epha5
Efnb3
Epha10
COX
.250
0
-0.250
Cancer
3 2
3*
3 2
2
2
*
-
-
3 2*
3 2
-
2
-
2
-
-
-
3
-
-
-
