# Supplementary material for: Diagnostic and Prognostic Markers for Pancreatitis and Pancreatic Ductal Adenocarcinoma
Source: Int J Mol Sci. 2024 Jun 16;25(12):6619. doi: 10.3390/ijms25126619 (PMC11204091; doi:10.3390/ijms25126619)
Supplement: Supplementary file 1 [file ijms-25-06619-s001.zip › Figure S9_KEGG and Wiki .pdf.zip!/SF9_KEGG and Wiki .pdf]

**SF9**

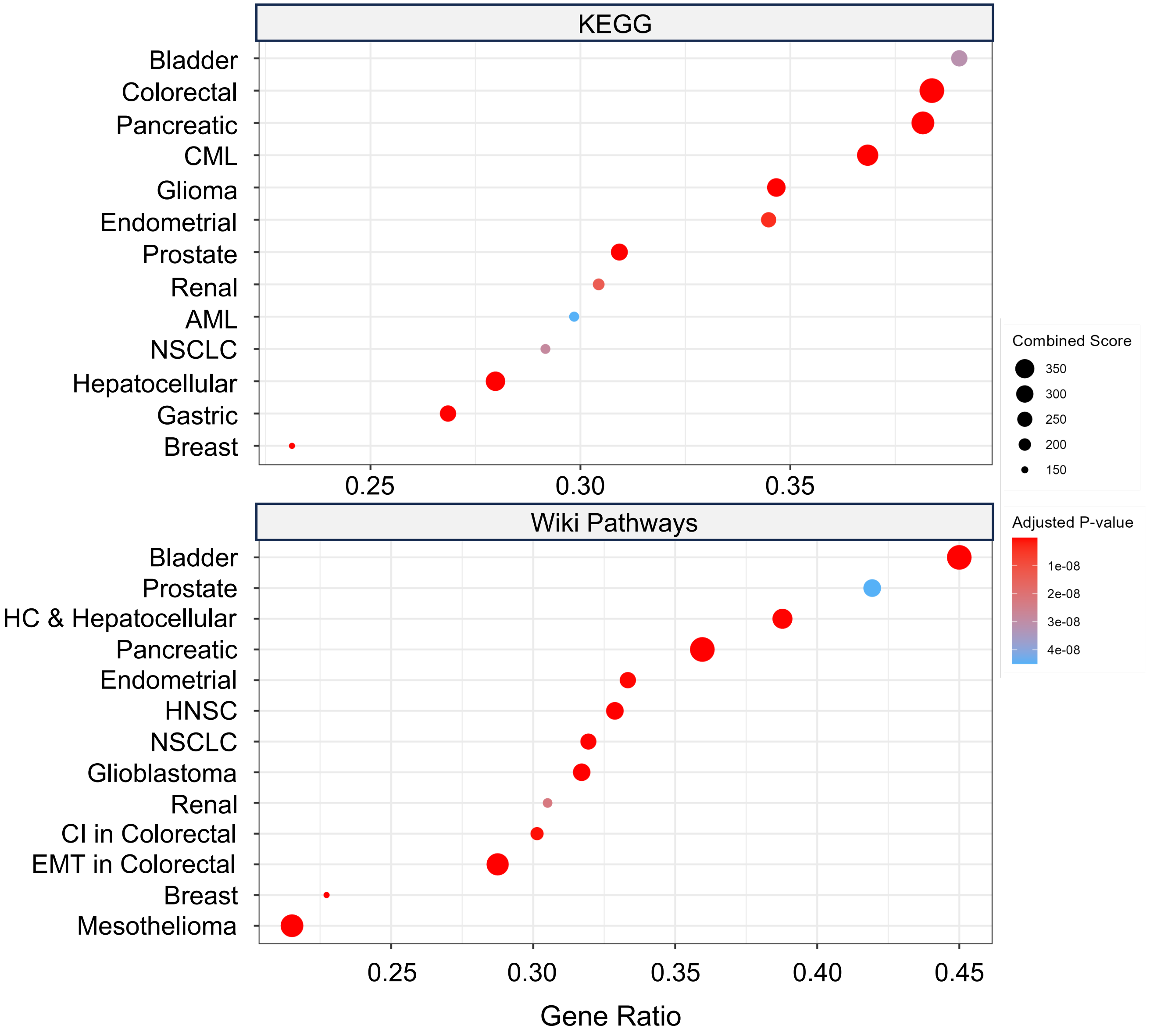
KEGG
Bladder
Colorectal
Pancreatic
CML
Glioma
Endometrial
Prostate
Renal
AML
NSCLC
Hepatocellular
Gastric
Breast
0.25
0.30
0.35
Wiki Pathways
Bladder
Prostate
HC & Hepatocellular
Pancreatic
Endometrial
HNSC
NSCLC
Glioblastoma
Renal
CI in Colorectal
EMT in Colorectal
Breast
Mesothelioma
0.25
0.30
0.35
0.40
0.45
Gene Ratio
Combined Score
350
300
250
200
150
Adjusted P-value
1e-08
2e-08
3e-08
4e-08
